# Proyecto VALIDA: Validation of ALlergy *In vitro* Diagnostics Assays (Herramientas y recomendaciones para la valoración de las pruebas *in vitro* en el diagnóstico de la alergia)

**DOI:** 10.1515/almed-2020-0022

**Published:** 2020-07-27

**Authors:** María L. Casas, Ángel Esteban, Miguel González-Muñoz, Moisés Labrador-Horrillo, Mariona Pascal, Aina Teniente-Serra

**Affiliations:** Sociedad Española de Medicina de Laboratorio (SEQC-ML), Barcelona, España; Servicio de Análisis Clínicos, Hospital Universitario Fundación Alcorcón, Alcorcón, Madrid, España; Servicio de Análisis Clínicos, Hospital General Universitario de Alicante, Alicante, España; Sociedad Española de Inmunología (SEI), Barcelona, España; Servicio de Inmunología, Hospital Universitario La Paz, Madrid, España; Sociedad Española de Alergología e Inmunología Clínica (SEAIC), Madrid, España; Servicio de Alergología, Hospital Universitari Vall d’Hebron, Barcelona, España; Servicio de Inmunología, CDB, Hospital Clínic de Barcelona, IDIBAPS, Universitat de Barcelona, Barcelona, España; Red de Investigación ARADyAL, Instituto Carlos III, Madrid, España; Servicio de Inmunología, LCMN, Hospital Universitari Germans Trias i Pujol, Badalona, España

**Keywords:** alergia, diagnóstico *in vitro*, inmunoglobulina E, recomendaciones

## Abstract

En la evaluación del paciente con sospecha de alergia las pruebas de detección y cuantificación de la inmunoglobulina E (IgE) específica *in vitro* se usan de manera habitual en los laboratorios clínicos para ayudar en el diagnóstico de la alergia. Actualmente existen diferentes alternativas comerciales para realizar estos ensayos, pero los resultados obtenidos por cada uno de ellos pueden variar, lo que condiciona el diagnóstico y el tratamiento que se le proporcionará al paciente. Con el fin de dar respuesta a los retos planteados por las diferencias entre las distintas técnicas para la determinación *in vitro* de la IgE específica, un grupo de expertos ha recogido en un documento una serie de recomendaciones sobre las implicaciones que puede tener el uso de una determinada técnica *in vitro* y el impacto en el manejo del paciente alérgico que suponen las diferencias entre las distintas técnicas. La lectura y el análisis de este documento de consenso ayudarán a entender las implicaciones que tiene el cambio de método de diagnóstico *in vitro* en el manejo del paciente con alergia, en su calidad de vida y en los costes socioeconómicos asociados a la enfermedad.

## Introducción

A raíz del descubrimiento de la inmunoglobulina E (IgE) y el posterior desarrollo del primer método de diagnóstico para su determinación *in vitro*, ha habido un antes y un después en el diagnóstico de la patología alérgica mediada por IgE [1].

La alergia es una enfermedad que presenta una elevada prevalencia [2], [3], provoca un deterioro de la calidad de vida del paciente y, según el caso, puede implicar unos costes elevados, tanto directos como indirectos, para el Sistema Nacional de Salud y la sociedad [4], [5].

En la actualidad, en el mercado español se comercializan diferentes técnicas de determinación *in vitro* de IgE total y específica (tIgE y sIgE, respectivamente). La diversidad en la oferta de tecnologías disponibles, la complejidad de la patología alérgica y el aumento de su prevalencia [6], [7], [8], [9] han conllevado la necesidad de establecer unas recomendaciones para evaluar de manera objetiva las metodologías disponibles en la actualidad [10].

El objetivo del presente documento es recopilar la evidencia sobre las distintas técnicas de diagnóstico *in vitro* actualmente disponibles, así como elaborar una serie de recomendaciones que ayuden a valorar cuál es la técnica de detección de IgE *in vitro* que mejor se ajusta a las necesidades de la práctica clínica habitual de cada centro sanitario y qué se debe tener en cuenta al plantearse un cambio.

## Metodología

Para la elaboración del presente documento se conformó un grupo de trabajo integrado por especialistas que desarrollan su actividad profesional en el área de diagnóstico de la inmunoalergología. El grupo de trabajo fue designado por las 3 sociedades principales implicadas en el diagnóstico de alergia: la Sociedad Española de Medicina de Laboratorio (SEQC-ML), la Sociedad Española de Inmunología (SEI) y la Sociedad Española de Alergología e Inmunología Clínica (SEAIC). Cada sociedad eligió a dos de sus afiliados como representantes de esta, formando un panel de 6 expertos.

Se elaboró una relación de cuestiones que responder en el documento, para la que se realizaron diversas búsquedas bibliográficas. Las búsquedas se centraron en los siguientes aspectos: 1) diferencias entre las técnicas de determinación *in vitro* de sIgE disponibles; 2) características de la técnica de detección de sIgE *in vitro* ideal; 3) recomendaciones de las principales guías en las que se aborda el diagnóstico de la patología alérgica, y 4) posibles consecuencias de un cambio de técnica en el manejo del paciente a todos los niveles [4], [11], [12], [13], [14], [15]. Con el fin de poder cuantificar la evidencia científica se realizó una búsqueda bibliográfica de las publicaciones existentes para cada una de las diferentes técnicas de detección de sIgE *in vitro* comercialmente disponibles en España. La búsqueda se llevó a cabo en la base de datos Medline usando los siguientes términos en inglés para cada una de las técnicas *in vitro*: (“Allergy AND Immunology” [Mesh] OR Allergy) AND (ImmunoCAP NOT ISAC), (“Allergy AND Immunology” [Mesh] OR Allergy) AND (ImmunoCAP AND ISAC), (“Allergy AND Immunology” [Mesh] OR Allergy) AND (Immulite), (“Allergy AND Immunology” [Mesh] OR Allergy) AND (Euroline), (“Allergy AND Immunology” [Mesh] OR Allergy) AND (Allergy Explorer-ALEX). No se aplicaron filtros adicionales a la búsqueda. Posteriormente, se eliminaron manualmente las publicaciones que no correspondían a los criterios de búsqueda mencionados y se recogió el número de publicaciones, por técnica y por año, desde la publicación del primer trabajo de cada técnica hasta la actualidad. Por último, se buscaron los ensayos clínicos completados en los que se han utilizado las distintas técnicas de diagnóstico *in vitro* analizadas en la base de datos clinicaltrials.gov, utilizando los términos “nombre de la técnica”, “condición o enfermedad alérgica”, y seleccionando los estudios completados. La búsqueda fue acotada entre el 1 de enero de 1989 (año en el que se comercializó el primer reactivo para la detección de tIgE y sIgE) y el 1 de octubre de 2019 (fecha en la que se preparó este documento).

Finalmente, se debatieron los puntos clave de acuerdo con los resultados de la búsqueda bibliográfica, que fueron contrastados con la experiencia práctica de los expertos, para así consensuar las recomendaciones incluidas en el presente documento.

## Importancia de las técnicas *in vitro* en el diagnóstico de la alergia

Según la guía de la Academia Europea de Alergia e Inmunología clínica, EAACI, [7], el protocolo de diagnóstico recomendado ante un paciente con sospecha de alergia empieza y se basa en una historia clínica completa, que dirige el posterior estudio de sensibilización, normalmente primero *in vivo* por prueba cutánea (*skin prick test* o SPT) y a continuación un estudio *in vitro* en suero.

El estudio alergológico *in vivo* centrado básicamente en las pruebas cutáneas es rápido y posee una gran sensibilidad y especificidad, pero a la vez presenta ciertas limitaciones que hacen necesario, en la mayoría de los casos, el uso combinado de técnicas *in vitro*. Entre las limitaciones de las pruebas cutáneas destacan, principalmente, la falta de estandarización y la variabilidad de los extractos que se utilizan, la posible subjetividad en la interpretación de los resultados (que afecta, por tanto, a la reproducibilidad), la infrecuente – aunque no descartable – posibilidad de inducir una reacción sistémica en el paciente alérgico, la imposibilidad de valorar componentes alergénicos recombinantes, y la necesidad de retrasar su implementación en pacientes con diversas afecciones cutáneas (dermografismo, dermatitis, urticaria) y en tratamiento con antihistamínicos u otros fármacos [16].

Debido a todos los aspectos mencionados anteriormente, el estudio *in vitro* ha ido adquiriendo una importancia creciente en la última década, al ampliar las posibilidades y la especificidad diagnóstica en el campo de la alergología, y disminuir los riesgos para el paciente derivados de una posible reactividad cruzada o una clínica no concordante con los datos de sensibilización obtenidos en el laboratorio [17].

Las aportaciones del diagnóstico molecular de la alergia han sido ampliamente documentadas durante las últimas décadas. En este sentido, el documento de consenso publicado por la WAO-ARIA-GA^2^LEN [10], sostiene que el diagnóstico *in vitro* desempeña un papel importante en 3 fases clave del diagnóstico de la alergia: 1) distinción entre sensibilización genuina y reactividad cruzada; 2) evaluación del riesgo de nuevas reacciones sistémicas en casos seleccionados de alergia a alimentos, y 3) identificación de los mejores pacientes candidatos a recibir inmunoterapia. Por este motivo, es necesario conocer las diferencias existentes entre las distintas técnicas *in vitro* respecto al diagnóstico.

Para entender mejor los conceptos más relevantes sobre las pruebas diagnósticas basadas en la detección de sIgE *in vitro*, es importante conocer los términos más habituales en el manejo de estas tecnologías [18]:

### Determinación de IgE *in vitro*

Cuantificación de la concentración de IgE sérica en una muestra de sangre de un individuo. Puede determinarse la tIgE, es decir, toda la carga de IgE del paciente independientemente de su especificidad, o sólo la sIgE frente a un determinado extracto o componente alergénico.

### Alérgeno

Es el antígeno capaz de inducir una respuesta inmunológica que conlleva la producción de anticuerpos de tipo IgE en un organismo predispuesto después del primer contacto, y posteriormente ocasionar una reacción antígeno-anticuerpo que puede desencadenar síntomas clínicos si existe una nueva exposición (reacción de hipersensibilidad tipo I, o alérgica). Son los antígenos de la respuesta alérgica.

### Extracto total

Un extracto alergénico total es una solución acuosa, glicerinada o un liofilizado de proteínas que proviene de la extracción (habitualmente acuosa) de una fuente alergénica completa (p. ej., cacahuete o polen de olivo).

### Componentes de alérgeno

Son los componentes individuales de una fuente alergénica que reaccionan con la sIgE. La mayoría son proteínas con capacidad tanto de inducir como de desencadenar una reacción alérgica. Así pues, una fuente alergénica (p. ej., un alimento o un polen) debe considerarse como una mezcla de diferentes “componentes” alergénicos.

### Diagnóstico resuelto por componentes (CRD, según sus siglas en inglés)

El diagnostico resuelto por componentes, también llamado diagnóstico molecular, detecta y cuantifica los niveles de anticuerpos sIgE para un determinado componente individual, lo que proporciona no sólo un diagnóstico mucho más preciso del paciente alérgico, sino también la posibilidad de evaluar el riesgo de nuevas reacciones e identificar los mejores candidatos para propósitos terapéuticos.

## Diferencias entre las técnicas de diagnóstico *in vitro* de la alergia

Las técnicas de detección *in vitro* de la sIgE se basan en la unión de un determinado alérgeno a una fase sólida o líquida, a la que se unirá la sIgE del paciente para dicho alérgeno, tras la incubación con el suero de éste. Las moléculas de IgE no específicas para el alérgeno en cuestión se eliminarán mediante un lavado. Posteriormente se incubará el complejo sIgE-alérgeno con un anticuerpo anti-IgE marcado que permitirá detectarlos. La señal emitida por el anticuerpo marcado permitirá medir la concentración de sIgE [19]. Los inmunoensayos para sIgE requieren una curva estándar de calibración para determinar la cantidad de IgE presente en el suero del paciente realizada a partir de unos calibradores concretos. Dichos calibradores para la IgE total están estandarizados respecto a la Preparación de Referencia Internacional para IgE Humana de la Organización Mundial de la Salud [20]. Esta se usa para interpolar resultados a kU_A_/L de sIgE, donde una unidad equivale a 2,4 ng de IgE. Existe evidencia de que una unidad de kU_A_/L de sIgE equivale a una unidad de kU/L de tIgE [21].

En los últimos años, los avances en la tecnología y la creciente importancia del diagnóstico *in vitro* de la alergia han dado lugar al desarrollo de varias técnicas, basadas en el sistema de detección de IgE descrito en el párrafo anterior, ya disponibles en el mercado español: ImmunoCAP™ e ImmunoCAP™ ISAC (Thermo Fisher Scientific), Immulite^®^ (Siemens), Euroline^®^ (Euroimmun) y ALEX^®^/ALEX^2^^®^ (Macro Array Diagnostics).

El análisis de las características de las distintas técnicas de diagnóstico *in vitro* de la alergia muestra que, pese a que todos los ensayos están basados en un reconocimiento antígeno-anticuerpo, éstos difieren en los métodos de unión a alérgenos, los métodos de detección de la señal, el volumen de muestra necesario, el tipo de cuantificación y el grado de automatización [22], [23], [24], [25]. El estudio alergológico *in vitro* se puede realizar mediante técnicas *singleplex*, que permiten la detección de niveles de sIgE frente a un único alérgeno en concreto o fuente alergénica, o *multiplex*, que consiguen medir la presencia de sIgE frente a una batería de alérgenos de manera simultánea [26].

A continuación, se especifican algunas características de cada una de ellas:

### Sistemas singleplex

El método ImmunoCAP™ presenta un catálogo de más de 600 alérgenos (entre los que se incluyen más de 100 componentes moleculares) para los que realiza una determinación cuantitativa (fluoroenzimoinmunoensayo).

Immulite^®^ es un método que dispone de más de 480 extractos alergénicos diferentes y 33 componentes moleculares. La tecnología utilizada es un enzimoinmunoensayo por quimioluminiscencia, con resultados cuantitativos.

### Sistemas multiplex

El ensayo *multiplex* ImmunoCAP™ ISAC utiliza una matriz fija de 112 componentes alergénicos recombinantes o nativos purificados fijados por triplicados, cuya determinación es semicuantitativa (fluorescencia).

El método ALEX^®^/ALEX^2^^®^ utiliza una matriz fija de más de 120 extractos alergénicos y 170 componentes moleculares; se obtienen resultados semicuantitativos para IgE total y cuantitativos para sIgE, mediante la tecnología de inmunoensayo en fase sólida (colorimetría).

Euroline^®^ es una técnica que ofrece la determinación de alrededor de 100 paneles distintos de alérgenos (que incluyen extractos totales y componentes), con resultados semicuantitativos mediante la tecnología de inmunoensayo en fase sólida (colorimetría).

En la Tabla 1 se incluye un resumen de las principales características de cada una de las técnicas.

**Tabla 1:** Comparativa entre las diferentes técnicas de diagnóstico *in vitro*.

Método de diagnóstico *in vitro*	Proveedor	Extractos completos	Componentes	Curva de calibración	Método de lectura	Proceso	Vol. (µL) [69], [70]	iCCD	Rango de detección [70]	Método/resultados
ImmunoCAP™	Thermo Fisher Scientific	>600	>100	Sí	Fluorimetría	Automatizado	40	No	0.10–100 kU_A_/L	*Singleplex*/cuantitativa
Immulite^®^	Siemens	>400	33	Sí	Quimioluminiscencia		50	No	0.10–100 kU_A_/L	
ImmunoCAP™ ISAC	Thermo Fisher Scientific	0	112	Sí	Fluorimetría	No automatizado	30	No	0.3–100 kU_A_/L	*Multiplex*/semicuantitativa
ALEX^®^	Macro Array Diagnostics	>120	>170	No	Colorimetría		100	Si^a^	sIgE: 0.3–50 kU_A_/L	*Multiplex*/IgE total semicuantitativa
									tIgE: 1–2500 kU/L	IgE específica cuantitativa
Euroline^®^	Euroimmun	92 paneles/desde 2 hasta 50 alérgenos por panel		No	Colorimetría		100–400	Si	0.35–100 kU_A_/L	*Multiplex*/semicuantitativa

Características de las diferentes técnicas de diagnóstico *in vitro* disponibles en el mercado español. Contenido de la tabla según las especificaciones de la ficha técnica del producto. Vol, volumen; iCCD, inhibición CCD. ^a^Dilución 1/5.

Una prueba diagnóstica debe ofrecer un balance óptimo desde el punto de vista de sensibilidad y especificidad, y disponer de evidencia científica que avale su uso. Además, sería conveniente que cubriera un amplio abanico de alérgenos y que permitiera la automatización. Crameri define el ImmunoCAP como la técnica *gold standard* de diagnóstico *in vitro* [27], a la espera de nueva evidencia científica respecto al resto de técnicas. Por otro lado, es la técnica de referencia con la que se comparan las demás en los estudios de correlación [28], [29], [30].

## Consideraciones ante un cambio de técnica *in vitro*

### Correlación entre las diferentes técnicas

La evidencia científica ha permitido comprobar que los resultados de las diferentes técnicas *in vitro* no son comparables ni intercambiables [23], [31], [32], [33]. Aunque en algunos casos podamos encontrar cierta correlación entre técnicas, los resultados no son intercambiables mediante el uso de factores de conversión, ya que, como se ha comentado anteriormente, no disponemos de unidades referidas a un estándar común [23], [33], [34], [35], [36], [37]. A modo de ejemplo, el estudio comparativo entre Immulite e ImmunoCAP llevado a cabo por Wood y col. [35] puso de manifiesto que no hay ningún método validado en el diagnóstico *in vitro* de la alergia que correlacione los resultados de las diferentes técnicas de forma fiable; asimismo, los datos obtenidos mediante una técnica no se pueden reproducir en otra técnica. Las repercusiones clínicas de la cuantificación de sIgE son importantes, por lo que el uso de uno u otro método es una decisión relevante.

### Fuentes de alérgenos: calidad y reproducibilidad

La calidad del alérgeno utilizado en cada técnica varía debido a varios factores, entre ellos, la temporada en la que se recoge la materia prima (p. ej., pólenes), el sistema de conservación del material, el grado de dificultad de su identificación, la contaminación con otras fuentes alergénicas que pueden dar lugar a una reactividad cruzada y las diferencias en la metodología de extracción (producción recombinante frente a purificación) entre los diferentes fabricantes [38]. Por todo ello, es necesario que los alérgenos sean sometidos a un exhaustivo y riguroso control de calidad.

También es importante conocer si el alérgeno contiene *cross-reactive carbohydrate determinats* (CCD) que son oligosacáridos presentes en muchos alérgenos con alta reactividad cruzada y escasa relevancia clínica, pudiendo dar lugar a falsos positivos. Con respecto a la prevalencia de estos antígenos y su impacto, se estima que estos pueden llegar a presentar reactividad en un 7,5–35% los pacientes, por lo que puede representar un problema a la hora del diagnóstico [39], [40], [41]. En la actualidad se aplican tres estrategias para evitar falsas positividades por CCDs: 1) producción recombinante de los alérgenos (en el caso de los componentes), 2) uso de inhibidores de CCDs durante el proceso de detección de sIgE o 3) uso de MUXF3 (alérgeno compuesto exclusivamente por epítopos carbohidrato presente en muchas glicoproteínas vegetales) como control de positividad por CCDs. Con el uso de estas estrategias se observa una disminución de los falsos positivos y, por lo tanto, un aumento de la precisión diagnóstica [41], [42], [43].

### Diversidad de catálogo

Es importante que el catálogo de alérgenos presente variedad tanto de extractos totales como de componentes moleculares, puesto que ambos son complementarios para un correcto diagnóstico. Es decir, saber a qué fuente alergénica estamos sensibilizados, y específicamente a cuál o cuáles de los distintos componentes alergénicos de esta fuente, es una información de gran valor clínico [44], [45].

En el día a día de la práctica clínica, disponer de un catálogo amplio puede contribuir a diagnosticar a más pacientes, ya que estamos ante una patología variable que puede resultar muy compleja en algunos casos [46]. Esto es especialmente relevante en nuestro entorno, ya que en España la polisensibilización representa un problema grave, igual que en otros países del entorno mediterráneo, como Italia y Grecia [47].

### Diferencias en la literatura científica

Valorar la evidencia científica disponible respecto a una metodología permite al profesional de la salud tomar decisiones más objetivas. Por eso es importante conocer las diferencias entre las distintas técnicas disponibles.

Antes de seleccionar un método para el diagnóstico *in vitro* de la alergia, es necesario asegurar que éste disponga de una evidencia científica suficiente proveniente de estudios, cuyo fin sea la mejora en el manejo del paciente alérgico. Lo contrario puede llevar a un retraso y una menor precisión en el diagnóstico, lo que podría repercutir en la calidad de vida del paciente.

**Figura 1:**
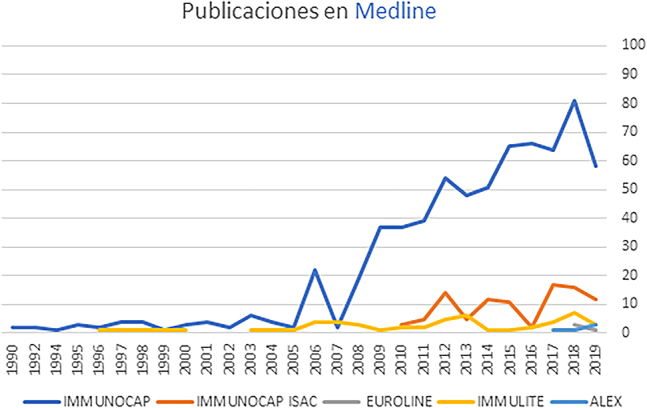
Gráfico de publicaciones en Medline por técnica de diagnóstico in vitro y por año.

Es por este motivo que se realizó una búsqueda de la literatura científica publicada sobre las distintas técnicas de detección de sIgE *in vitro*. Los resultados se detallan a continuación:

Siguiendo el orden cronológico de aparición de la evidencia de cada técnica de diagnóstico *in vitro* de alergia, y utilizando los criterios de búsqueda descritos anteriormente (ver apartado de metodología), se observa que en el momento en que se redactó este documento ImmunoCAP (1990) presenta más de 600 publicaciones en la base de datos Medline, Immulite (1996) ha sido referenciado en 52 artículos indexados, ImmunoCAP ISAC (2010) aparece en 117 artículos, Euroline (2018) en 7 artículos, y ALEX (2018) es la técnica utilizada en 3 publicaciones (Tabla 2, Figura 1). Debido al impacto de la localización geográfica en los perfiles de sensibilización, más allá de valorar el número de publicaciones, también es muy importante disponer de evidencia local: ImmunoCAP e ImmunoCAP ISAC son las técnicas que disponen de una mayor evidencia hasta el momento (Material Suplementario, Tabla 1). ImmunoCAP e ImmunoCAP ISAC disponen de más de 45 y 12 estudios completados en España, respectivamente. Immulite se ha utilizado en 2 estudios nacionales (Material Suplementario, Tablas 1 y 2).

**Tabla 2:** Comparativa de las diferentes técnicas *in vitro* en función de la evidencia científica disponible.

Técnica	No total de publicaciones en Medline	Tasa de publicación^a^	Estudios clínicos (total de pacientes incluidos)
ImmunoCAP^®^	633	22,1	22 (2.381)
Immulite^®^ 2000	52	2,16	1 (102)
ImmunoCAP ISAC^®^	117	13	0
Euroline^®^	7	1,4	1 (235)
Allergy Explorer-ALEX^®^	3	1,5	0

El número de estudios clínicos corresponde a los que se aplican a la enfermedad alérgica y que ya están completados y publicados en https://clinicaltrials.gov.

^a^Tasa de publicación: total de publicaciones/años desde la primera publicación.

### Impacto del cambio de método de diagnóstico *in vitro*

En la valoración del impacto del cambio de método de determinación de sIgE *in vitro* siempre hay que considerar que una prueba positiva sólo indica sensibilización, y sólo si está asociada a reactividad clínica (síntomas) podemos hablar de alergia [48]. No existen puntos de corte universales para los diferentes alérgenos que permitan predecir la probabilidad de reactividad clínica asociada a un determinado valor de sIgE, por lo que éstos se deben validar clínicamente para cada técnica. Si un laboratorio cambia de técnica, sería necesario volver a establecer los valores de referencia, ya que los anteriores no serán válidos. En este sentido, es importante que haya comunicación con el clínico para establecer cómo se va a hacer la transición y establecer nuevos valores de referencia. Por tanto, tras el cambio de una técnica por otra, será necesario revisar cómo los resultados obtenidos se aplican en la práctica clínica diaria [23], [29], [32].

### Impacto en el diagnóstico inicial del paciente

Las diferencias entre métodos diagnósticos pueden afectar a la interpretación de los resultados y causar confusión con respecto a los protocolos establecidos en los laboratorios y servicios clínicos que manejan al paciente alérgico. Por ejemplo, los valores de corte utilizados para las pruebas de provocación oral propuestos a partir de la experiencia acorde a los resultados de una técnica de diagnóstico *in vitro* determinada, no son válidos para las distintas técnicas para su uso en la toma de decisiones clínicas. Así, las pruebas de provocación con alimentos se basan en puntos de corte locales; cada centro utiliza puntos de corte propios que, en caso de que cambie la metodología, deberían establecerse de nuevo [23]. Esto tiene diversas implicaciones: aumento de los costes asociados al diagnóstico, molestias para el paciente, riesgos para la seguridad del paciente y sobrecarga del sistema de salud.

### Impacto en la toma de decisiones terapéuticas

Hoy en día, se considera que el único tratamiento capaz de modificar el curso natural de la enfermedad alérgica es la desensibilización o la inmunoterapia específica con alérgeno (ITA). En el caso de la ITA, se ha demostrado que puede prevenir la progresión de la enfermedad, y su efecto terapéutico perdura incluso tras haber finalizado su administración [49]. Como su nombre indica, la ITA es específica para un extracto alergénico determinado, por lo que la identificación del alérgeno causante de la enfermedad es esencial para la prescripción más adecuada del tratamiento.

Por este motivo, la determinación de sIgE frente a extracto total, complementado con el diagnóstico *in vitro*, puede mejorar la selección del paciente para la indicación de ITA; por ejemplo, en la alergia a pólenes [50], [51] y al veneno de himenópteros [52]. Por tanto, es aconsejable disponer de un único método que permita la realización en paralelo de componentes y extractos totales, para poder diferenciar a los pacientes alérgicos con múltiples sensibilizaciones genuinas de aquellos con multisensibilización por reactividad cruzada [53], sobre todo en los países con alta prevalencia de pacientes polisensibilizados [54]. Además, el diagnóstico *in vitro* puede permitir evaluar el riesgo de reacciones adversas a la inmunoterapia con algunas fuentes alergénicas, como el polen de olivo en zonas de alta exposición polínica, ya que la sensibilización a determinados componentes alergénicos (p. ej., a Ole e 9 y Ole e 7), está asociada a este tipo de reacciones [55], [56].

### Impacto en el seguimiento

El cambio de técnica de diagnóstico *in vitro* durante el seguimiento de un paciente alérgico y pendiente de revisión puede suponer un problema más importante que el que representaría para el paciente diagnosticado por primera vez. Esta afirmación coincide con la literatura disponible, en la que se aconseja que, debido a la variabilidad entre Immulite y otros ensayos de sIgE, es preferible usar un solo ensayo para controlar la evolución de la alergia [28], [34], [37], [51].

Otros ejemplos los podemos encontrar en los casos en el que el seguimiento del paciente implica la realización de prueba de tolerancia a alimentos tras un periodo de evitación, según unos determinados puntos de corte de sIgE, después del diagnóstico de alergia alimentaria [57].

### Impacto en el coste asociado de la enfermedad alérgica

En la alergia, como en cualquier patología, tanto a la hora de elegir un método diagnóstico como un tratamiento, se evalúa fundamentalmente su relación coste-efectividad. Esto conlleva que, en el proceso de adquisición de un método, los criterios que se tienen en cuenta son tanto técnicos como económicos (estos últimos normalmente limitados al precio unitario de la prueba). Esta aproximación, dentro del marco de la contratación pública, permite a los profesionales de la salud valorar cuál de los métodos disponibles aporta la mejor relación coste-efectividad.

Es importante que los especialistas que trabajan en el área de la inmunoalergología conozcan en profundidad las características técnicas de cada una de las metodologías disponibles, así como las diferencias entre ellas, lo que permitirá entender las implicaciones de un cambio de técnica y los costes derivados de ello, tanto directos como indirectos [58], [59].

Hasta la fecha, los estudios de coste-efectividad publicados se centran en el rendimiento de ImmunoCAP ISAC y SPT. En 2016 el NICE analizó en una revisión sistemática 4 estudios que las incluían [60]. En diversos trabajos de Hermanasen y col (2012, 2013) se compara el coste efectividad del ImmunoCAP ISAC frente provocación oral controlada con doble ciego y el SPT en niños con alergia a los cacahuetes. Para ello se usó un modelo de Markov a 5 años, siendo el ImmunoCAP ISAC el más eficiente frente a la provocación y el SPT [61], [62]. Glaumann y colaboradores (2013) también estudiaron el coste efectividad del ImmunoCAP ISAC frente a la provocación oral y el SPT, mostrando de nuevo ImmunoCAP dominancia sobre el resto de técnicas [63]. Por otra parte Hermansson y col. (2012) [64] y Mascialino y col. (2013) [65] analizaron el coste efectividad de ImmunoCAP ISAC frente a SPT en una población alérgica al polen, usando un modelo de Markov con un horizonte a 9 años y observaron que la suma de ImmunoCAP ISAC y SPT reduce la prescripción de inmunoterapia frente a la STP sola.

## Discusión

Hasta la fecha, la legislación europea de productos sanitarios y a diferencia de la Food and Drug Administration, no exige a los productos sanitarios una validación mediante ensayos clínicos realizados en pacientes previa a la comercialización [66], diferenciándose de esta manera de los fármacos. El único requisito para su comercialización en Europa es la obtención del marcado CE. Para esto se requiere al fabricante presentar un estudio de comparación con otro método y demostrar que es reproducible en el tiempo. Dicha comparación debe aparecer en las instrucciones de uso del producto. En este sentido, es importante tener en cuenta que el estudio *in vitro* de detección de sIgE presenta particularidades relacionadas con la naturaleza de la IgE respecto al estudio de otros parámetros analíticos. Por este motivo, resulta importante conocer bien las técnicas de diagnóstico *in vitro* de detección de sIgE a la hora de decidir la mejor opción.

Por otra parte, se ha descrito que un diagnóstico impreciso tiene consecuencias en distintos ámbitos, con su correspondiente impacto económico:

Laboratorio. Hay que considerar el coste y el tiempo necesarios para realizar una validación clínica exhaustiva de una nueva técnica de diagnóstico *in vitro*. Como ya se ha explicado anteriormente, la validación necesaria para la comercialización de un producto de diagnóstico *in vitro* no requiere la realización de ensayos clínicos con pacientes. Esta situación normativa implica que es responsabilidad del propio laboratorio y, por extensión, de todos los implicados en el diagnóstico del paciente alérgico, diseñar y ejecutar una validación clínica que tenga en cuenta la realidad poblacional para verificar que el nuevo método de diagnóstico para detección de IgE cumple con los requisitos necesarios, tanto de panel de alérgenos como de sensibilidad y especificidad analíticas.
Clínico. Un cambio de método diagnóstico puede dar pie a la obtención de resultados no concluyentes, si no se establecen los nuevos valores de referencia, lo que hace necesario el uso de más recursos para contrastar los resultados. Esto conllevaría un aumento en el gasto, tanto en número de determinaciones como en dedicación por parte del clínico.
Logístico. Si la nueva técnica seleccionada no incluye en su portafolio los alérgenos necesarios y previamente utilizados, será necesario en muchos casos externalizar la determinación de sIgE, con el correspondiente coste asociado y la posible demora de los resultados en el tiempo.

Aunque este trabajo se ha centrado en los costes directos, también sería necesario valorar los posibles efectos en la calidad de vida de los pacientes, así como los costes indirectos que, aunque no se han descrito en este apartado, también pueden ser considerables [56].

Por otra parte, cabe destacar que la falta de estudios de comparación *head to head* entre las distintas técnicas ha sido una limitación a la hora de elaborar este manuscrito. Además, no tan solo faltan comparativas *head to head*, sino también literatura en general para muchas de las técnicas comercializadas más recientemente. Es por este motivo que se dispone de escasas herramientas para valorar un cambio de técnica y por ende un manuscrito de estas características era necesario. Otras limitaciones en el campo de la alergología, que dificultan la valoración de la prueba de detección de sIgE *in vitro* más adecuado para cada laboratorio, son la variabilidad de los paneles de alérgenos en el caso del diagnóstico molecular, la falta de estandarización de los alérgenos y por tanto su reproducibilidad y la falta de controles de calidad. [67], [68].

## Conclusiones

En la actualidad existen distintas técnicas para la determinación de sIgE *in vitro*, y es importante conocer sus características para establecer un correcto diagnóstico y manejo del paciente alérgico.
Tanto las técnicas *singleplex* como las *multiplex* son útiles en el diagnóstico alergológico, y su uso dependerá de la complejidad de cada caso y de las necesidades específicas del paciente.
La evidencia científica disponible y la experiencia acumulada en la práctica clínica habitual permiten afirmar que los resultados de los diferentes métodos comercializados para el diagnóstico alergológico *in vitro* no son intercambiables; por tanto, es importante mantener el mismo método de detección de sIgE durante el diagnóstico, el tratamiento y el seguimiento del paciente.
Cualquier cambio de método diagnóstico *in vitro* implica ineludiblemente realizar una validación exhaustiva por parte del laboratorio y los especialistas que controlan la patología alérgica del paciente.
Un cambio de técnica puede tener implicaciones en el manejo del paciente alérgico que impacten negativamente en distintos niveles, además de suponer un incremento de costes asociados, tanto directos como indirectos.
Una técnica de calidad es la que presenta un balance correcto entre sensibilidad y especificidad analítica, dispone de evidencia científica suficiente que avale su utilidad clínica, tiene un amplio catálogo de alérgenos y es coste-efectiva.

En la actualidad, a falta de más evidencia respecto a las tecnologías más novedosas, ImmunoCAP es la técnica que se ajusta mejor a los aspectos valorados en este documento, y que aporta mayor aval científico para el diagnóstico de la alergia *in vitro* en la población general.

## Supplementary Material

Supplementary Material Details

Supplementary Material Details

## Material Supplementario

La versión en línea de este artículo ofrece material suplementario (https://doi.org/10.1515/almed-2020-0022).
