# Supplementary material for: Proyecto VALIDA: Validation of ALlergy In vitro Diagnostics Assays (Herramientas y recomendaciones para la valoración de las pruebas in vitro en el diagnóstico de la alergia)
Source: Adv Lab Med. 2020 Jul 27;1(4):20200022. [Article in Spanish] doi: 10.1515/almed-2020-0022 (PMC10197503; doi:10.1515/almed-2020-0022)
Supplement: Supplementary file 1 — Supplementary Material Details [file j_almed-2020-0022_suppl_001.docx]

**Material complementario**

**Tabla 1.** Estudios clínicos en alergia con ImmunoCAP (completados)

| Código NTC | Titulo | Condición | Reclutamiento (n) | Localización |
| --- | --- | --- | --- | --- |
| NCT02328170 | Comparative Study of Specific IgE Levels to Common Foods and Aeroallergens Measured by Euroimmun Allergy and ImmunoCAP | Alergia | 235 | Tailandia |
| NCT02666092 | Anisakis Blastocystis Cryptosporidium Fish Serology | Alergia al pescado | 105 | Francia |
| NCT01264601 | Safe Administration of Flu Vaccine to Egg Allergic Children | Alergia al huevo | 31 | Estados Unidos |
| NCT00715156 | Role of Recombinants in Peach Allergy | Alergia alimentaria | 148 | Italia |
| NCT01634737 | Crustacean Allergy and Dust Mites Sensitization | Alergia alimentaria | 100 | Italia |
| NCT01641731 | Specific Oral Tolerance Induction in Children Allergic to Cow’s Milk Proteins | Alergia a la leche | 55 | España |
| NCT01792232 | Effects of Co-Exposure to Air Pollution and Allergen | Alergia | 18 | Canadá |
| NCT02979600 | Clinical and Biological Efficacy of Peanut Oral Immunotherapy | Alergia al cacahuete | 493 | Francia |
| NCT02644785 | Serum Tryptase Levels During Cardiac Surgery, Diagnosis and Treatment Decisions for Allergic Reactions | Alergia a la protamina | 30 | Turquía |
| NCT01589731 | Polymerized Beta-lactoglobulin Comparative Immunoreactivity | Alergia a la leche | 114 | Brasil |
| NCT01489553 | Egg Oral Immunotherapy | Alergia al huevo | 9 | Estados Unidos |
| NCT00597675 | Oral Immunotherapy for Peanut Allergy (PMIT) | Hipersensibilidad alimentaria | 10 | Estados Unidos |
| NCT03048149 | Clinical and Biological Efficacy of Hazelnut Oral Immunotherapy | Alergia | 100 | Francia |
| NCT01552161 | Prevalence of Allergic Diseases and Atopy in Subjects With Coronary Artery Disease | Alergia | 300 | Polonia |
| NCT02382718 | FAST Fish Phase IIb Clinical Trial for the Treatment of Fish Allergy by Subcutaneous Immunotherapy | Alergia al pescado | 45 | Dinamarca, Grecia, Islandia, Holanda, Polonia, España |
| NCT01007253 | Effect of Veramyst and Olopatadine 0.2% Ophthalmic Solution on Allergy Symptoms | Rinitis alérgica estacional | 21 | Estados Unidos |
| NCT01966224 | A Safety and Immunogenicity Phase IB Study of CryJ2-DNA-Lysosomal Associated Membrane Protein (CryJ2-DNA-LAMP) Plasmid Assessing the Long-Term Safety of Previously Treated Subjects | Rinoconjutivitis alérgica | 17 | Estados Unidos |
| NCT02486159 | The Oligonucleotide Chip Analysis for Allergic Rhinitis Treatment in Herbal Plaster and Acupuncture | Rinitis | 50 | China |
| NCT00346398 | Promoting Tolerance to Common Allergens in High-Risk Children: Global Prevention of Asthma in Children (GPAC) Study | Asma | 51 | Estados Unidos y Australia |
| NCT01644617 | A Dose-Ranging Study of the Safety and Effectiveness of MK-8237 in the Treatment of House Dust Mite (HDM) Induced Allergic Rhinitis/Rhinoconjunctivitis in Adults (MK-8237-003/P07627) | Rinitis alérgica perenne | 124 | Austria |
| NCT02733016 | Seinäjoki Adult Asthma Study | Asma | 259 | Finlandia |
| NCT01942096 | Study of Airway Inflammation in Relation to Exercise in Elite Athletes | Asma inducida por ejercicio | 66 | Bélgica |
