# Supplementary material for: Proyecto VALIDA: Validation of ALlergy In vitro Diagnostics Assays (Herramientas y recomendaciones para la valoración de las pruebas in vitro en el diagnóstico de la alergia)
Source: Adv Lab Med. 2020 Jul 27;1(4):20200022. [Article in Spanish] doi: 10.1515/almed-2020-0022 (PMC10197503; doi:10.1515/almed-2020-0022)
Supplement: Supplementary file 2 — Supplementary Material Details [file j_almed-2020-0022_suppl_002.docx]

**Material Complementario**

**Tabla 2.** Estudios intervencionales con ImmunoCAP (Thermo Fisher) completados

| Alergia | Código NTC | (n) total | Localización |
| --- | --- | --- | --- |
| Huevo | NCT01264601 | 31 | Estados Unidos |
| Rinitis alérgica | NCT02146781 | 22 | Estados Unidos |
| Rinoconjuntivitis | NCT01966224 | 17 | Estados Unidos |
| Rinitis alérgica | NCT02486159 | 50 | Taiwán |
| Sensibilidad a las proteínas de la leche de vaca | NCT01641731 | 55 | España |
| Alergia | NCT01792232 | 18 | Canadá |
| Asma | NCT00346398 | 51 | Estados Unidos/Australia |
| Hipersensibilidad a alimentos | NCT00597675 | 10 | Estados Unidos |
| Rinitis alérgica temporal | NCT01007253 | 21 | Estados Unidos |
| Rinitis alérgica perenne | NCT01644617 | 124 | Austria |
| Alergia al pescado | NCT02382718 | 45 | Dinamarca, Grecia, Islandia, Holanda, Polonia, España |
